# 3D organoid cultivation improves the maturation and functional differentiation of cholangiocytes from human pluripotent stem cells

**DOI:** 10.3389/fcell.2024.1361084

**Published:** 2024-07-08

**Authors:** Nova Yuli Prasetyo Budi, Wei-Yu Lai, Yen-Hua Huang, Hong-Nerng Ho

**Affiliations:** ^1^ International Ph.D. Program in Cell Therapy and Regenerative Medicine, College of Medicine, Taipei Medical University, Taipei, Taiwan; ^2^ Department of Biochemistry and Molecular Cell Biology, School of Medicine, College of Medicine, Taipei Medical University, Taipei, Taiwan; ^3^ Pediatric Surgery Division, Department of Surgery/Genetics Working Group, Faculty of Medicine, Public Health and Nursing, Universitas Gadjah Mada/Dr. Sardjito Hospital, Yogyakarta, Indonesia; ^4^ TMU Research Center for Cell Therapy and Regeneration Medicine, Taipei Medical University, Taipei, Taiwan; ^5^ Graduate Institute of Medical Sciences, College of Medicine, Taipei Medical University, Taipei, Taiwan; ^6^ Department of Obstetrics and Gynecology, School of Medicine, College of Medicine, Taipei Medical University, Taipei, Taiwan; ^7^ Department of Obstetrics and Gynecology, Taipei Municipal Wanfang Hospital, Taipei Medical University, Taipei, Taiwan; ^8^ Taipei Cancer Center, Taipei Medical University, Taipei, Taiwan

**Keywords:** cholangiocyte differentiation, human pluripotent stem cells, organoids, cholangiopathy, epidermal growth factor receptor signaling

## Abstract

Idiopathic cholangiopathies are diseases that affect cholangiocytes, and they have unknown etiologies. Currently, orthotopic liver transplantation is the only treatment available for end-stage liver disease. Limited access to the bile duct makes it difficult to model cholangiocyte diseases. In this study, by mimicking the embryonic development of cholangiocytes and using a robust, feeder- and serum-free protocol, we first demonstrate the generation of unique functional 3D organoids consisting of small and large cholangiocytes derived from human pluripotent stem cells (PSCs), as opposed to traditional 2D culture systems. At day 28 of differentiation, the human PSC–derived cholangiocytes expressed markers of mature cholangiocytes, such as *CK7*, *CK19*, and cystic fibrosis transmembrane conductance regulator (*CFTR*). Compared with the 2D culture system–generated cholangiocytes, the 3D cholangiocyte organoids (COs) showed higher expression of the region-specific markers of intrahepatic cholangiocytes *YAP1* and *JAG1* and extrahepatic cholangiocytes *AQP1* and *MUC1*. Furthermore, the COs had small-large tube-like structures and functional assays revealed that they exhibited characteristics of mature cholangiocytes, such as multidrug resistance protein 1 transporter function and *CFTR* channel activity. In addition to the extracellular matrix supports, the epidermal growth factor receptor (EGFR)-mediated signaling regulation might be involved in this cholangiocyte maturation and differentiation. These results indicated the successful generation of intrahepatic and extrahepatic cholangiocytes by using our 3D organoid protocol. The results highlight the advantages of our 3D culture system over the 2D culture system in promoting the functional differentiation and maturation of cholangiocytes. In summary, in advance of the previous works, our study provides a possible concept of small-large cholangiocyte transdifferentiation of human PSCs under cost-effective 3D culture conditions. The study findings have implications for the development of effective cell-based therapy using COs for patients with cholangiopathies.

## Introduction

Cholangiopathies comprise a diverse group of disorders characterized by damage to and loss of cholangiocytes, which are specialized epithelial cells that line the interconnected bile ducts; these disorders can result in cholestasis, hepatic injury, and, eventually, liver failure (Park, 2012; Lazaridis and LaRusso, 2015; Banales et al., 2019). No effective medical treatment exists for cholangiopathies; therefore, they usually progress to end-stage liver disease, for which the only treatment option is liver transplantation (Babu et al., 2020). Cholangiopathies are indicated in approximately 10%–16% of all liver transplantations (Lazaridis and LaRusso, 2015; Burra et al., 2016). Research on cholangiopathies is hindered because there are limited cells and animal models available (Sampaziotis et al., 2015b; Fabris et al., 2019), making it challenging to conduct in-depth studies and develop alternative therapies (De Assuncao et al., 2017). A recent study showed that primary human cholangiocyte organoids could resolve cholangiopathy in the liver under normothermic machine perfusion (Sampaziotis et al., 2021) but access to original human cholangiocytes is limited. This poses challenges in establishing high-throughput cholangiocyte organoids and may impede clinical translation efforts (Ogawa et al., 2021).

Human induced pluripotent stem cells (iPSCs) possess capabilities similar to human embryonic stem cells (ESCs), such as the capacity for self-replication and differentiation into nearly all cell types, including cholangiocytes. As a result, human iPSCs serve as a promising resource for the development of cholangiocyte organoids used in drug testing, *in vitro* disease modeling, and cell therapies (Shi et al., 2017). A recent study emphasizes the value of iPSCs in elucidating novel genetic and epigenetic mechanisms that underlie the intricate relationship between genotype and phenotype (Rossi and Kontarakis, 2022). The differentiation of human iPSCs into cholangiocytes is regulated by several signaling pathways, including Notch, transforming growth factor-β (TGF-β), fibroblast growth factor (FGF), bone morphogenetic protein (BMP), Ca^2+^ and Wnt signaling pathways (Clotman et al., 2005; Minagawa et al., 2006; Bogert and LaRusso, 2007; Decaens et al., 2008; Yanai et al., 2008; Tchorz et al., 2009; Lemaigre, 2010; Banales et al., 2019; Lemaigre, 2020). Moreover, the Wnt signaling pathway also plays a crucial role in the cholangiocyte transdifferentiation (Hussain et al., 2004; Decaens et al., 2008; Graffmann et al., 2018; Verstegen et al., 2020). Cholangiocytes can be generated *in vitro* from human PSCs by manipulating these signaling pathways. Different differentiation protocols have been applied to generate mature cholangiocytes from human ESCs and iPSCs by using two (2D) or three-dimensional (3D) culture systems (Dianat et al., 2014; Sampaziotis et al., 2015a; Ogawa et al., 2015; Takayama et al., 2016; Sampaziotis et al., 2017a; Matsui et al., 2019; Luce and Dubart-Kupperschmitt, 2020; Ogawa et al., 2021). To enhance the functionality and maintenance of human PSC–derived cholangiocytes, approaches such as extracellular matrices, transcription factor overexpression, and modified cultivation media using chemically defined medium have been suggested (Kamiya and Chikada, 2015).

Despite advances in protocols, challenges still exist regarding the differences in the maturation and plasticity of generated mature cholangiocytes. Sampaziotis group has developed a protocol to create cholangiocytes that mimic intrahepatic cholangiocytes from the human PSCs (Sampaziotis et al., 2015a; Sampaziotis et al., 2017a) and isolation of primary cholangiocytes from extrahepatic biliary tree (Sampaziotis et al., 2017b). Other studies have used coculture with OP9 cells to generate intrahepatic cholangiocytes in the canals of Hering (Dianat et al., 2014; Ogawa et al., 2015; Ogawa et al., 2021). Up to date, there is no 2D monolayer or 3D culture system has been reported to generate intrahepatic or extrahepatic cholangiocytes. Furthermore, the potential signaling pathway responsible for cholangiocyte transdifferentiation *in vitro* remains unknown.

To advance the previous works, this study successfully established a robust, feeder- and serum-free, and cost-effective protocol for the 2D and 3D differentiation of mature cholangiocytes from human PSCs. These protocols were refined based on prior studies (Dianat et al., 2014; De Assuncao et al., 2015; Sampaziotis et al., 2015a; Ogawa et al., 2015; Takayama et al., 2016; Sampaziotis et al., 2017a; Matsui et al., 2019; Luce and Dubart-Kupperschmitt, 2020; Ogawa et al., 2021; Jalan-Sakrikar et al., 2022). The unique 3D culture platform can generate mature cholangiocytes with high plasticity, forming tube-like structures and facilitating small-large cholangiocyte transdifferentiation. This was confirmed through the evaluation of differentiation using core biliary markers (*CK7*, *CK19,* and *CFTR*), region-specific markers of intrahepatic cholangiocytes (*YAP1* and *JAG1*), and specific markers of extrahepatic cholangiocytes (*AQP1* and *MUC1*), as well as functional assays assessing multidrug resistance protein 1 transporter function and CFTR channel activity. The potential involvement of EGF-Wnt signaling in the regulation of differentiation was suggested. Additionally, the study discussed the advantages of 3D culture systems over 2D systems in enhancing cholangiocyte maturation and functionality. These findings hold promise for contributing to the development of future cell-based therapies for cholangiopathies.

## Experimental procedures

### Human pluripotent stem cells

The iPSC cell lines of iNFB3 and IBMS-iPSC-01-02 (IB0102) were obtained from Professor Hong-Nerng Ho’s laboratory at National Taiwan University Hospital (Huang et al., 2019). The cells were periodically frozen and thawed while being continuously maintained in ReproCELL serum-free medium (ReproCELL, Shinyokohama, Yokohama, Japan). The cells were passaged weekly by using 30-gauge insulin needles (Terumo, Shinjuku, Tokyo, Japan) (Chen et al., 2015). iNFB3 is an iPSC cell line derived from human dermal fibroblasts that were episomally reprogrammed into human iPSCs by using electroporation to introduce *OCT4*, *SOX2*, *KLF4*, and *c-MYC*, as described previously (Chen et al., 2015). IBMS-iPSC-01-02 (IB0102) is an iPSC cell line derived from human peripheral blood mononuclear cells reprogrammed using Sendai virus vectors to transiently drive the expression of *OCT4*, *SOX2*, *KLF4*, and *c-MYC*. A human ESC line (H9; karyotype 46, XX; WiCell, WI, United States) (Thomson et al., 1998) was also obtained from Professor Hong-Nerng Ho’s laboratory at National Taiwan University Hospital. These cells were periodically frozen and thawed while being continuously maintained using the same protocol as that for human iPSCs (Chen et al., 2015).

### Cholangiocyte differentiation

The protocol and detailed materials information on cholangiocyte differentiation from the human pluripotent stem cells in this study were provided in Supplementary Tables S1, S2. In brief, the cholangiocyte differentiation protocol developed in this study was optimized on the basis of previous studies (Dianat et al., 2014; Sampaziotis et al., 2015a; Ogawa et al., 2015; De Assuncao et al., 2015; Takayama et al., 2016; Sampaziotis et al., 2017a; Matsui et al., 2019; Luce and Dubart-Kupperschmitt, 2020; Ogawa et al., 2021; Jalan-Sakrikar et al., 2022). In this protocol, 28 days are required to differentiate mature cholangiocytes from human PSCs. Induction of human PSC–derived definitive endoderm (DE) cells was performed by adding a high concentration of Activin A in stage 1 (days 1–4). Subsequently, DE cells were induced to differentiate into ventral foregut endoderm (VFE) in stage 2 (days 5–8). In stage 3 (days 9–13), VFE was differentiated into hepatoblasts (HBs), which are bipotent progenitors of hepatocytes and cholangiocytes (i.e., capable of developing into either hepatocytes or cholangiocytes). In stage 4 (days 14–17), HBs committed to the biliary lineage, and cholangiocyte progenitors (CPs) were generated as early cholangiocytes. In the final stage, 3D culture system was utilized to generate mature cholangiocytes from CPs until day 28 of the differentiation.

### Quantitative real-time polymerase chain reaction

Total RNA was isolated using RNeasy Mini Kit (QIAGEN, Valencia, CA, United States) following the manufacturer’s guidelines. The concentration of total mRNA was assessed using a microvolume spectrophotometer (NanoPhotometer N60, Implen, Munich, Germany). mRNA was then reverse transcribed into cDNA by using Moloney Murine Leukemia Virus (MMLV) reverse transcriptase (Invitrogen, Carlsbad, CA, United States), followed by polymerase chain reaction (PCR) amplification by using the Fast SYBR Green Master Mix (Thermo Fisher Scientific, Waltham, MA, United States) on a real-time (RT) PCR system (LightCycler 96, Roche Diagnostics, Basel, Switzerland). The amplification process comprised initial denaturation at 95°C for 30 s, followed by 35 cycles of denaturation at 95°C for 3 s and annealing/extension at 60°C for 30 s. The GAPDH gene served as an internal control. The primer sequences and experimental conditions employed in this study are detailed in Supplementary Table S3.

### Alkaline phosphatase activity assay

Human iPSCs and ESCs were cultured for 4–5 days after passaging and were then evaluated for alkaline phosphatase activity. Subsequently, cells were fixed with 4% paraformaldehyde (Sigma-Aldrich, St. Louis, MO, United States) for 2 min. Alkaline phosphatase activity in both human iPSCs and ESCs was analyzed using an alkaline phosphatase detection kit following the manufacturer’s guidelines (Merck Millipore, Darmstadt, Germany).

### Flow cytometry

Cells were treated with Triple Select Enzyme (1X) without phenol red (Gibco, Grand Island, NY, United States) for 5 min to facilitate their dissociation into single cells. Following neutralization with cell medium, cells were harvested and washed with phosphate-buffered saline containing 5% fetal bovine serum (Gibco). Cell blocking was conducted by incubation with 10% fetal bovine serum for 30 min, followed by incubation with the primary antibody at 37°C for 50 min. Cells were washed twice in phosphate-buffered saline, resuspended, exposed to the secondary antibody for an additional 20 min, and then again washed twice in phosphate-buffered saline. For intracellular markers, cells were fixed with 4% paraformaldehyde (Sigma-Aldrich) for 10 min, permeabilized with 0.5% Triton X 100 for 20 min, incubated with the primary antibody for 50 min, and then incubated with the secondary antibody for 20 min. The stained cells were assessed using a flow cytometer (FACSVerse, BD, San Jose, CA, United States) and quantified using FlowJo (version 10.5.3, Becton Dickinson). Details regarding the antibodies and their experimental conditions are summarized in Supplementary Table S4.

### Immunofluorescence (IF) microscopy assay

The 2D and 3D culture system–generated cholangiocyte organoid (CO) samples were fixed with 4% paraformaldehyde (Sigma-Aldrich). After fixation, the 2D culture system-generated cholangiocytes were permeabilized for 10 min using 0.1% Triton X 100 containing 1% bovine serum albumin (Sigma-Aldrich), and the 3D culture system–generated CO samples underwent the same permeabilization treatment for 30 min. All samples were then blocked with 3% bovine serum albumin and incubated with the primary antibody overnight at 4°C. On the following day, the samples were washed with phosphate-buffered saline (Sigma-Aldrich) and exposed to the secondary antibody for either 1 h (for 2D culture system–generated cholangiocytes) or 2 h (for 3D culture system–generated CO samples). After washing, the samples were stained with DAPI for an additional 10 min and then stored using an antifade-added cover glass or in a chambered dish to preserve the organoid structure of the cells for subsequent confocal microscopy. CO images were captured using a confocal microscope (STELLARIS 8, Leica Microsystems, Wetzlar, Germany) employing Z-stacking to generate 3D images. The antibodies and experimental conditions employed in this study are detailed in Supplementary Table S4.

### Western blot analysis

Cell extracts were prepared using lysis buffer (10 mM Tris, pH 7.5; 150 mM NaCl; 1 mM EDTA; 0.5% sodium deoxycholate; 0.5% NP-40; and 0.1% SDS) supplemented with a protease inhibitor cocktail (Roche Diagnostics) and phosphatase inhibitor cocktail (Roche Diagnostics). The protein concentration was determined using an assay kit (Pierce BCA, Thermo Fisher Scientific). Equivalent amounts of total protein were separated through 10%–12% sodium dodecyl sulfate–polyacrylamide gel electrophoresis and were subsequently transferred to nitrocellulose membranes (Immobilon-P PVDF Membrane, Merck Millipore). These PVDF membranes were then blocked with 5% bovine serum albumin and incubated with specific primary antibodies. Signal detection was accomplished using a biomolecular imager (ImageQuant LAS 4000 mini, GE Healthcare, Uppsala, Sweden). Western blot analysis was performed in accordance with a previous protocol (Peng et al., 2023). The obtained Western blot image was subjected to quantification using the ImageJ software (Schneider et al., 2012). Details regarding the antibodies and their experimental conditions are summarized in Supplementary Table S4.

### Rhodamine-123 functional study

To investigate the presence of calcium channels, with a focus on multidrug resistance protein 1B (MDR1b) transporter on COs, the CO samples were placed into a chambered dish. Each sample was pretreated with dimethyl sulfoxide (Sigma-Aldrich) and verapamil (ChemScene, New Jersey, United States) in William’s E medium (Gibco) for 20 min and then washed twice in William’s E medium. The samples were then treated with Rhodamine 123 (ChemScene) for 30 min, then washed in William’s E medium (Gibco), and observed using a confocal microscope. Obtained images were subjected to quantification using ImageJ software (Schneider et al., 2012).

### Forskolin-induced swelling assay

The COs were treated with forskolin (10 µM) to evaluate CFTR function in the COs. The dimethyl sulfoxide (DMSO) was used in the control group. The COs were incubated with forskolin for specific durations up to 120 min. Residual forskolin was then removed by a careful washing process, and the abundance, size, and morphology of the COs were examined under a light microscope. Images were taken for quantitative analysis.

### Statistical analysis

Data are presented as mean ± standard error of the mean (SEM), except where indicated otherwise. The data were analyzed using unpaired Student’s t-test or one-way ANOVA, followed by *post hoc* analysis for pairwise comparisons between groups. Data were analyzed using SPSS (IBM) and visualized using GraphPad Prism 8. Statistical significance was indicated by *p* < 0.05. Livak (2^−ΔΔCT^) method was utilized to determine the genes expression (Livak and Schmittgen, 2001).

## Results

### Cultivation and characterization of human pluripotent stem cells

**FIGURE 1:**
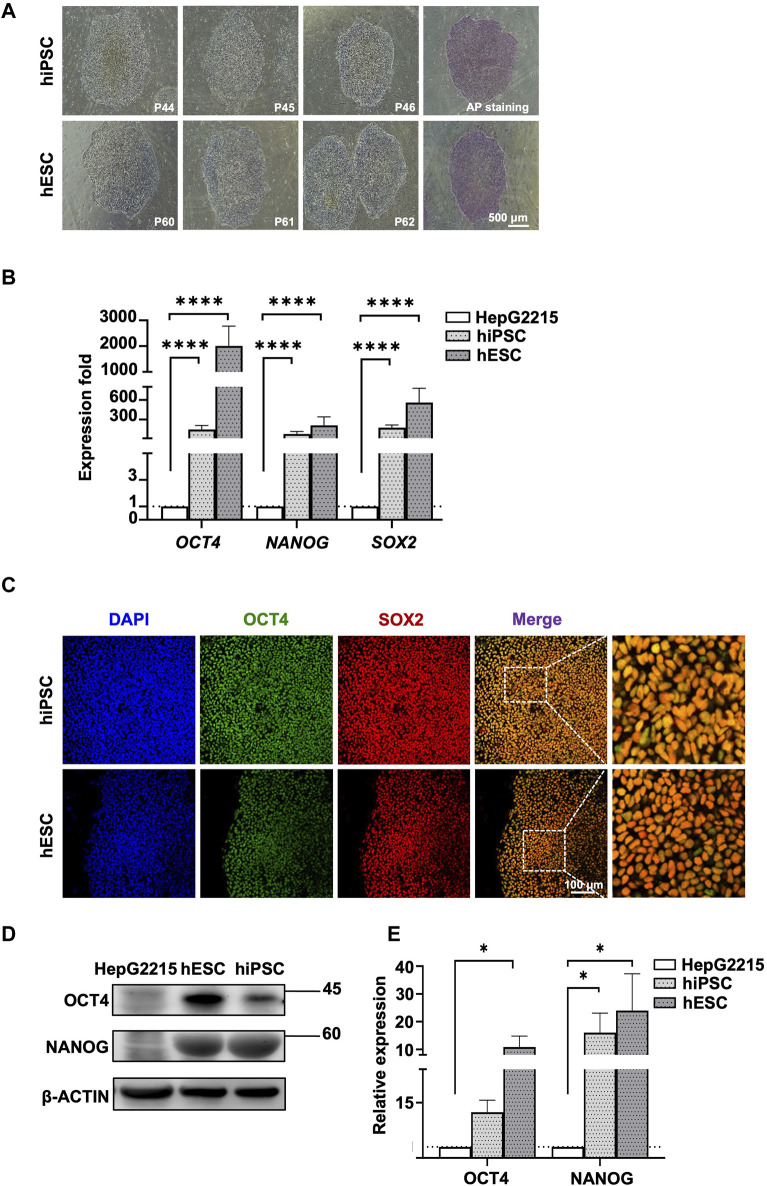
Characterization of human iPSCs and ESCs. **(A)** Morphology of human iPSCs and ESCs. Human ESC and iPSC colonies were positively stained with AP. **(B)** Expression fold of pluripotent genes *OCT*, *NANOG*, and *SOX2* in human PSCs. Gene expression was normalized to *GAPDH* expression and HepG2215 cell line. **(C)** Immunofluorescence staining revealed positive pluripotent markers, including OCT4 and SOX2, in human iPSC and ESC. The nuclei of all cells were stained blue with DAPI. **(D)** Western blot analysis revealed increases in OCT4, NANOG, and SOX2 expression in human PSCs with statistically significant relative expression. **(E)** The bar chart shows protein quantification differences, including OCT4, NANOG, and SOX2, in human PSCs compared to HepG2215. Protein quantification was normalized to corresponding β-actin in Figure 1; Data are presented as mean ± SEM in **(B,E)**; *p* values were determined using one-way ANOVA from at least three independent experiments; *p < 0.05, **p < 0.01, ***p < 0.001, ****p < 0.0001; P, passage number; AP, alkaline phosphatase.

The human PSCs used in this study were iPSCs and ESCs. Both the iPSCs and ESCs presented colony morphology and strong alkaline phosphatase activity (Figure 1A). Further analysis using RT quantitative PCR (qPCR) revealed that the expression of the pluripotent transcription factors *OCT4*, *NANOG*, and *SOX2* was significantly higher in both the human PSCs than in a somatic liver cancer cell line (HepG2215; Figure 1B). Protein concentrations were analyzed through IF staining (Figure 1C) and Western blotting (Figure 1D), with quantitative analysis provided in Figure 1E.

### Generation of DE and VFE from human PSCs

**FIGURE 2:**
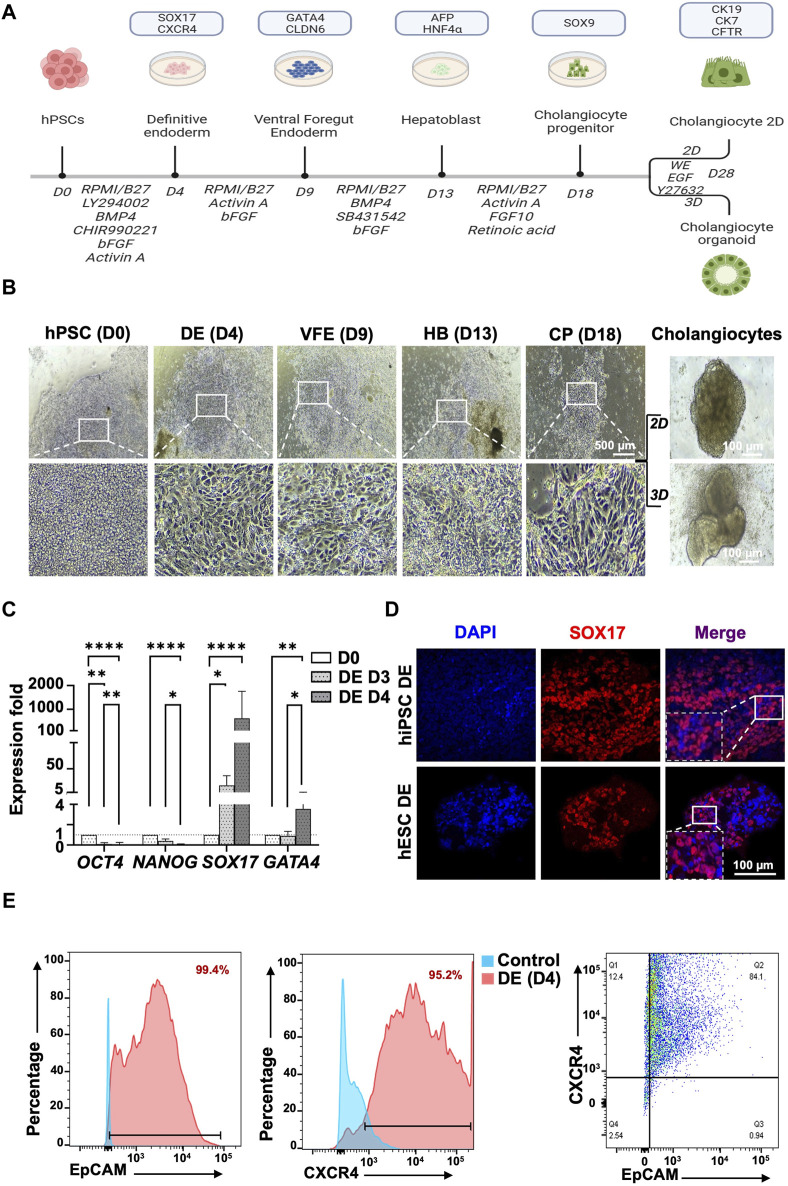
Protocol for generating human pluripotent stem cell-derived cholangiocytes from human PSCs and definitive endoderm stage characterization. **(A)** The protocol proceeds through the phases of definitive endoderm (DE), ventral foregut endoderm (VFE), hepatoblast (HB), cholangiocyte progenitor (CP), and mature cholangiocyte. **(B)** Light microscopy revealed morphological changes in each stage of differentiation with the transformation from stem cell phenotype toward an epithelial 2D of cholangiocyte progenitor cells and subsequent maturation into cholangiocytes by using 2D and 3D culture systems. 3D culture generated mature cholangiocytes with tube-like morphology. **(C)** Characterization of definitive endoderm (DE) stage at day 4 of differentiation. qPCR revealed higher expression of DE markers, *SOX17,* and *GATA4* in 4-day than in 3-day cultures. **(D)** Immunofluorescence staining revealed that at day 4 of differentiation, cells expressed DE marker SOX17. **(E)** Flow cytometry revealed that 99.4% of cells were EpCAM^+^ and 95.2% of cells were CXCR4^+^ at day 4 of differentiation. Furthermore, 84.1% of cells were both EpCAM^+^ and CXCR4^+^. Data are presented as mean ± SEM in **(C)**; *p* values were determined using one-way ANOVA from at least three independent experiments; *p < 0.05, **p < 0.01.

To generate mature cholangiocytes from human PSCs, a 28-day novel induction protocol was established (Figure 2A). The human PSCs were differentiated into primitive streak, mesoderm, and DEs by using Activin A, Ly294002 (an inhibitor of phosphoinositide 3-kinase [PI3K]) and CHIR99021 (an inhibitor of glycogen synthase kinase 3β [GSK3β]). A morphological change and the epithelial-mesenchymal transition revealed the first-stage differentiation of human PSCs into DE (Figure 2B, human PSC to DE). The primary DEs were continuously induced with a high concentration of Activin A and basic FGF (bFGF) until days 3 and 4. RT-qPCR analysis revealed that by days 3 and 4, the expression of the pluripotent transcription factor *OCT4* decreased and that the expression of the DE marker *SOX17* increased (Figure 2C). At day 4, the expression of SOX17 protein was determined using IF staining (Figure 2D). Successful DE differentiation was further confirmed using flow cytometry, which revealed the double-positive signals of EpCAM^+^ (99.4%) and CXCR4^+^ (95.2%; Figure 2E). An additional surface marker was also employed, the c-KIT (92,7%; Supplementary Figure S1).

**FIGURE 3:**
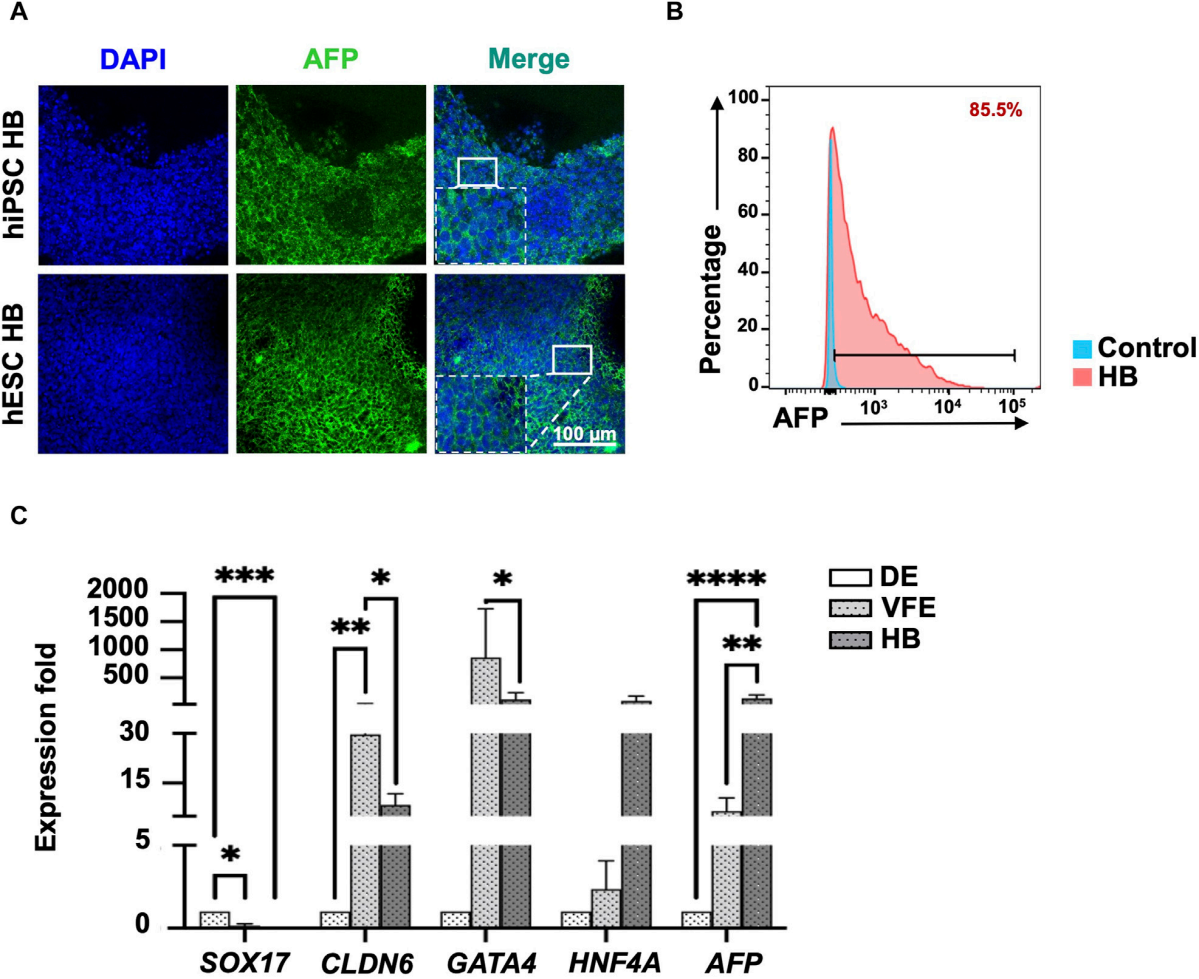
Characterization of hepatoblast (HB) stage at day 13 of differentiation. **(A)** Immunofluorescence staining revealed that HBs expressed HB marker AFP. **(B)** Flow cytometry revealed that 85.5% of cells were AFP^+^ at the end of the HB stage. **(C)** qPCR revealed that the expression of HB marker *AFP* had significantly increased, and endoderm markers expression, including *SOX17*, *CLDN6*, and *GATA4,* had significantly decreased at the HB stage. Data are presented as mean ± SEM in **(C)**; *p* values were determined using independent *t*-test from at least three independent experiments; *p < 0.05.

The differentiation of DE into VFE was induced using Activin A, a TGF-β superfamily member that determines the cell fate in respiratory and digestive tissues. Moreover, introducing FGF signaling will also enhance the differentiation of DE into VFE. By the end of day 9, cells had formed confluent epithelium, and cells also exhibited a characteristic of rhomboidal morphology (Figure 2B for hPSC iNFB3, Supplementary Figure S2 for hPSC IB0102 and H9) and significantly increased the VFE marker *GATA4* (*p* = 0.026) as revealed by RT-qPCR (Figure 3C, Supplementary Figure S3).

### Generation of bipotent HBs

For the differentiation of VFE into HB, the FGF signaling pathway was activated by adding bFGF, which synergistically acted with BMP and Wnt/β-catenin signaling. SB431542 was also added to inhibit the TGF-β pathway and promote HB differentiation (Figure 2A, stage 3: VFE–HB). Cell proliferation decreased, indicating HB differentiation (Figure 2B for hPSC iNFB3, Supplementary Figure S2 for hPSC IB0102 and H9). Concentrations of alpha-fetoprotein (AFP) in the differentiated HBs were determined using IF staining (Figure 3A) and flow cytometry at day 13, with more than 85% positive signals (Figure 3B). The expression of specific markers in the DE, VFEs, and HBs was examined using RT-qPCR. At the end of the VFE stage, we observed a significant increase in the expression of VFE-related genes, such as *CLDN6* and *GATA4*, accompanied by a notable decrease in the expression of the DE-associated gene *SOX17*. *HNF4A* and *AFP* expression was significantly higher in the HBs than in the DE and VFEs (Figure 3C, Supplementary Figure S3). Together, these results highlight the successful differentiation of VFEs into HBs under our culture protocol.

### Generation of CPs

**FIGURE 4:**
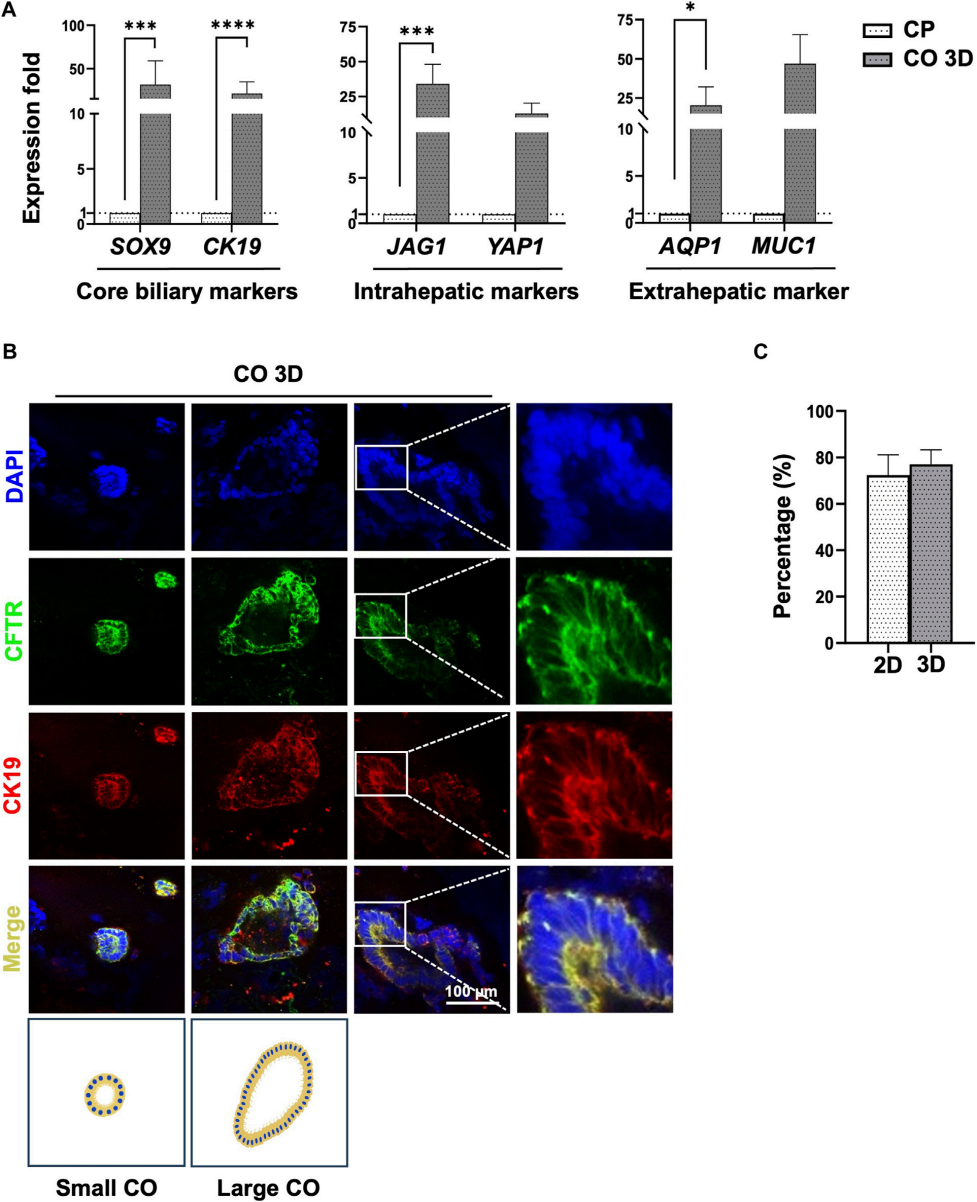
Characterization of cholangiocyte organoids from human PSCs at day 28 of differentiation. **(A)** Comparison of core biliary markers (*SOX9*, *CFTR*), intrahepatic (*JAG1*, *YAP1*), and extrahepatic (*AQP1*) cholangiocyte markers in cholangiocyte organoids (COs) and cholangiocyte progenitors (CPs). **(B)** COs were stained to detect core cholangiocyte markers CK19 and CFTR. Positive results were obtained, and COs had a tube-like form. Additional image illustrations of small- and large cholangiocyte organoids were shown. **(C)** Immunofluorescence signal quantification of CFTR/DAPI ratio revealed that cells generated using a 3D culture system had a higher percentage of CFTR-positive cells. Data are presented as mean ± SEM in **(A,C)**; *p* values were determined using an independent *t*-test from at least three independent experiments on **(A)** and two independent experiments on **(C)**. Data are presented as percentage ±SEM of CFTR/DAPI positive cells in **(C)**. Positive cell quantification was performed using ImageJ (Schneider et al., 2012; Shihan et al., 2021); *p < 0.05, **p < 0.01, ***p < 0.001, ****p < 0.0001.

To differentiate the bipotent HBs into CPs, the Notch and FGF signaling pathways were activated by the addition of Activin A, retinoic acid, and FGF10 (Figure 2A, stage 4: HB–CP). Cell proliferation was greater in the CPs than in the HBs (Figure 2B, day 18). The expression of the early cholangiocyte marker *SOX9* was determined using RT-qPCR (Figure 4A, Supplementary Figure S3), and IF staining (Supplementary Figure S4A). The 2D culture system–generated CPs expressed the mature cholangiocyte markers CK19 and EpCAM at day 28 (Supplementary Figure S4B). At this time, the HBs were still present in the culture dishes, indicating that the time of inducing the differentiation of HB into CP is a crucial factor of effective biliary commitment.

### Generation of functional COs using 3D culture system

Several studies have reported that activating Notch signaling can promote cholangiocyte maturation and inhibit hepatocyte differentiation (Tanimizu and Miyajima, 2004; Pasqua et al., 2021). However, we did not observe the formation of tube-like structures or functional cholangiocytes under a similar 2D culture condition (Figure 2B; Cholangiocytes stage).

To generate mature COs, CPs were dissociated into small clumps and cultivated using a 3D culture system. The size of cell clumps in the Matrigel dome and the EGF-supplemented culture medium used in the 3D culture system have critical effects on the efficiency of CO generation. The cell clumps turned into small or large cholangiocytes, forming tube-like structures within 10–15 days of cultivation (Figure 2B; Cholangiocyte stage). The expression of mature cholangiocyte markers and region-specific cholangiocyte markers was determined using RT-qPCR (Figure 4A, Supplementary Figure S5A). Importantly, the generated COs also exhibited the expression of region-specific markers of extrahepatic cholangiocytes (*AQP1* and *MUC1*) and intrahepatic cholangiocytes (*YAP1* and *JAG1*; Figure 4A). The expression levels of each target gene throughout the differentiation process are shown in Supplementary Figure S3.

The expression of the mature cholangiocyte markers CFTR and CK19 in small or large cholangiocytes was determined using IF staining (Figure 4B, Supplementary Figures S5B, S5C). CO generation efficiency was evaluated using the percentage of cells that had a normalized CFTR/DAPI ratio of 72.33% ± 8.7 for the 2D culture system and 77.12% ± 6.2% for the 3D culture system on day 28 (Figure 4C). The COs were further analyzed using 3D reconstruction in the Leica LAS X software package, showing the hollow tube-like organoid (Supplementary Videos S1, S2).

### Comparison of CO under 2D and 3D culture system

**FIGURE 5:**
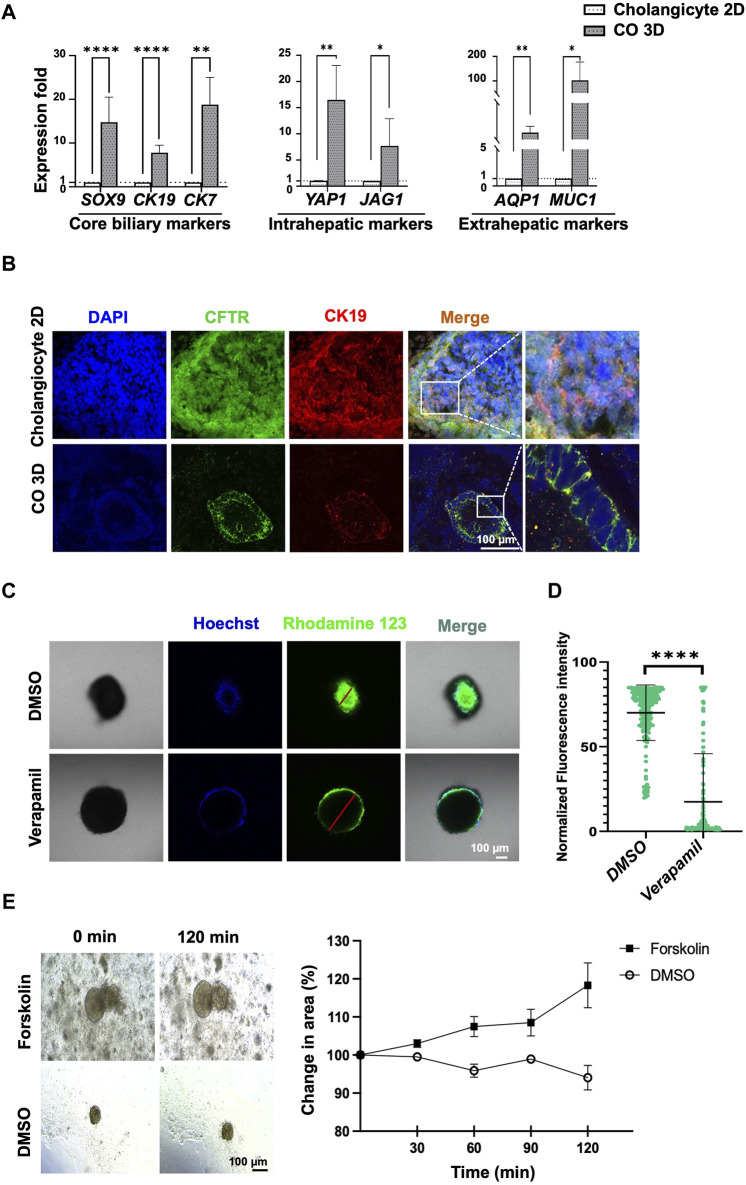
Comparison of mature cholangiocytes generated using 2D and 3D culture systems. **(A)** qPCR revealed that cholangiocytes generated using 3D culture system yielded higher expression of mature cholangiocyte markers and region-specific markers for cholangiocytes. **(B)** Immunofluorescence staining revealed differences between mature cholangiocytes generated using 2D versus 3D culture systems. **(C)** Functional analysis using rhodamine 123 revealed an accumulation of rhodamine inside the cholangiocyte cyst and an absence of accumulation in the verapamil group. The difference in fluorescence intensity was significantly higher. **(E)** Functional analysis using Forskolin-induced swelling revealed the percentage of the organoid area had increased after treatment with 10 µM of Forskolin. Data are presented as mean ± SEM in **(A,D)**; *p* values were determined using independent *t*-test from at least three independent experiments; Data presented on the **(E)** were calculated by measuring the longest diameter of the organoids from a single experiment; *p < 0.05, **p < 0.01, ***p < 0.001, ****p < 0.0001.

To compare the maturation of generated-COs under 2D and 3D cultures, we evaluated the core biliary markers and region-specific markers of cholangiocytes at day 28 of differentiation. The expression of mature cholangiocyte markers was higher in the 3D culture system–generated cholangiocytes than in the 2D culture system–generated cholangiocytes (Figure 5A). Further, IF staining analysis also revealed the tube-like structure of COs, a formation unattainable in a 2D culture system (Figure 5B). With long-term cultivation, the 3D culture system–generated organoids formed a branching tubular structure after passage (Supplementary Figure S6A), but their proliferative capacity will decrease starting from day 55–60 after passage under prolonged culture evaluated by IF staining for Ki67 (Supplementary Figure S6B).

### Generated CO under 3D culture system is functional

To determine the functionality of the 3D culture system–generated human PSC–derived COs, the function of P-glycoprotein was examined using rhodamine 123 and forskolin-induced swelling assay. The accumulation of rhodamine 123 within the lumen was indicative of P-glycoprotein activity, which in turn indicated the presence of mature and functional cholangiocytes (Figures 5C, D). Verapamil was used to block the MDR1 transporter; this blocking was confirmed by the absence of accumulation of fluorescence in the cytoplasm of the cholangiocytes (Figures 5C, D, Supplementary Figure S5D).

Furthermore, the CFTR function of the cholangiocyte 3D organoids was further assessed through a rapid and quantitative water-uptake swelling assay induced by forskolin (Ramalho et al., 2022). As depicted in Figure 5E, forskolin was observed to enhance organoid size compared to the DMSO control group. These results strongly demonstrated the successful generation of functional COs.

### Potential EGF/EGFR mediated signaling pathway in cholangiocyte transdifferentiation

**FIGURE 6:**
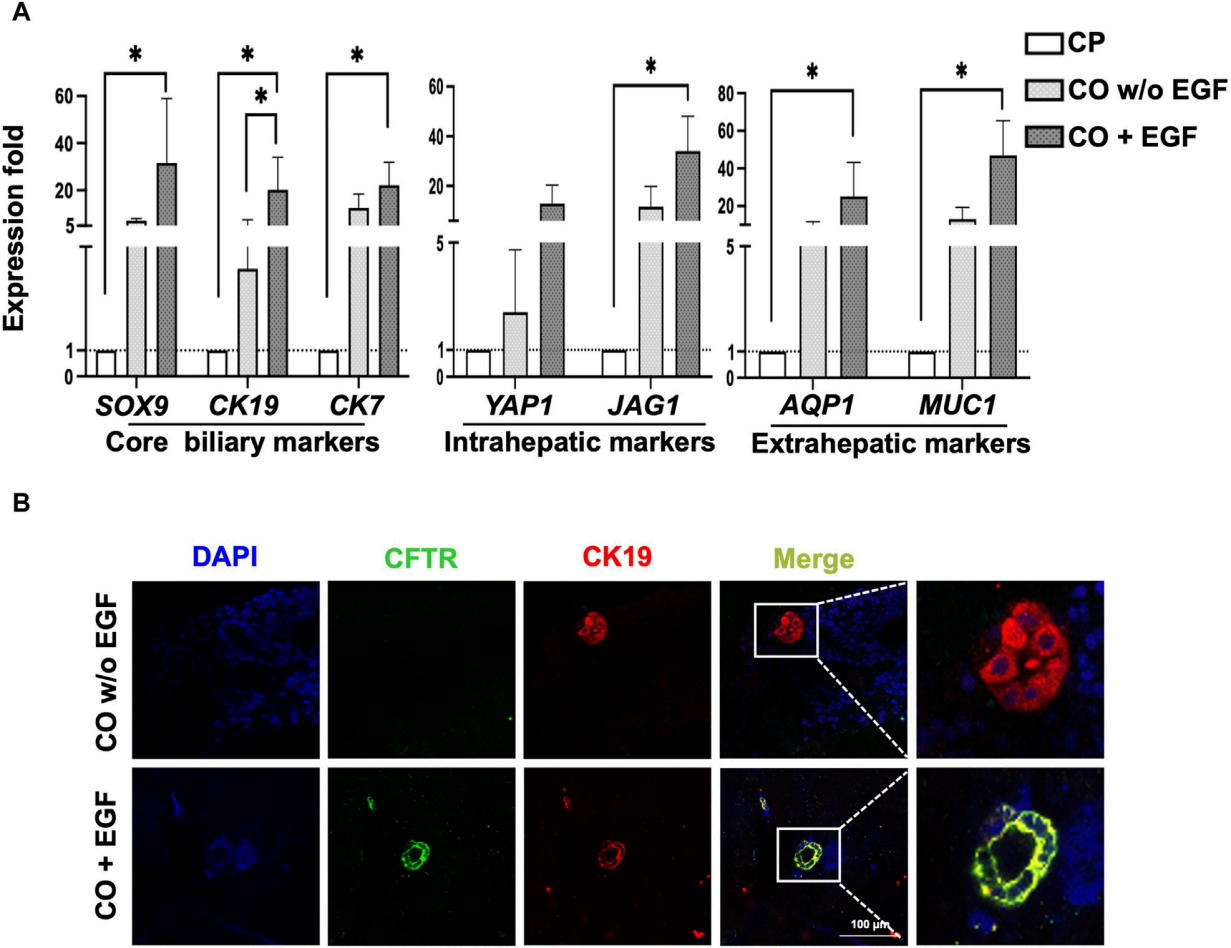
Characterization of COs under EGFR signaling conditions. **(A)** qPCR revealed that the EGF group had higher expression of overall core biliary markers and region-specific markers compared to the EGF-absence group. **(B)** Evaluation of COs using immunofluorescence staining for CFTR and CK19 revealed tube-like morphology in the EGF group. Data are presented as mean ± SEM in **(A)** from two independent experiments; *p < 0.05.

To examine the potential underlying mechanism in cholangiocyte transdifferentiation, EGF was used in this study as EGF has been known to regulate liver development and regeneration (McLin et al., 2007). We found that under EGF-absent conditions, the expression of the core biliary markers *CK19*, *CK17*, and *CFTR* increased, and after EGF treatment, the expression of the extrahepatic cholangiocyte markers *AQP1* and *MUC1* significantly increased (Figure 6A).

Further evaluation of core biliary markers, CFTR and CK19, using IF staining confirmed that the addition of EGF enhanced the expression of CFTR, as demonstrated by the 3D cholangiocyte morphology and improved protein expressions of CFTR and CK19 (Figure 6B). Additionally, our observations revealed that EGF significantly increased the expression of Wnt signaling target genes, including both canonical (*LGR5*) and non-canonical (*NKCC1*), as well as the biliary progenitor marker *NOTCH2* (Supplementary Figure S7). These preliminary findings suggest a potential correlation between EGF and Wnt signaling, supported by the upregulation of both canonical and non-canonical Wnt signaling target genes, along with the biliary progenitor gene.

## Discussion

To address the challenges presented by the high expenses and restricted accessibility of primary human cholangiocytes associated with generating cholangiopathies, our research has successfully developed robust, feeder- and serum-free, economical protocols for deriving mature cholangiocytes from human pluripotent stem cells, eliminating the requirement for a costly chemically defined medium. This advancement holds significant promise for cost-effective applications in future clinical settings. Additionally, we have enhanced the 3D reconstruction process for cholangiocyte organoids, resulting in the formation of tube-like structures encompassing both small and large cholangiocytes. Furthermore, the crucial role of epidermal growth factor (EGF) in promoting the maturation of cholangiocyte organoids has been emphasized.

This study represents the first to demonstrate the generation of unique functional 3D organoids composed of small and large cholangiocytes derived from human pluripotent stem cells, as opposed to traditional 2D culture systems. Through the utilization of a 3D culture system, we successfully produced cholangiocytes exhibiting tube-like morphology (Figures 4, 5). Our findings revealed the presence of both small and large mature cholangiocyte organoids within the 3D culture system (Figure 4). These 3D cholangiocyte organoids demonstrated enhanced expression of core biliary markers (*CK7*, *CK19*, and *CFTR*; Figure 5A), region-specific markers for extrahepatic cholangiocytes (*AQP1* and *MUC1*; Figure 5A), and region-specific markers for intrahepatic cholangiocytes (*YAP1* and *JAG1*; Figure 5A). Moreover, the generated cholangiocyte organoids exhibited positive functional activity, as evidenced by P-glycoprotein activity using rhodamine 123 staining (Figures 5C,D, Supplementary Figure S5D) and CFTR activity assessed through Forskolin-induced swelling assay (Figure 5E). Notably, this study not only produced functional cholangiocyte organoids but also did so in a cost-effective manner, offering promising implications for the future clinical management of cholangiopathies.

**FIGURE 7:**
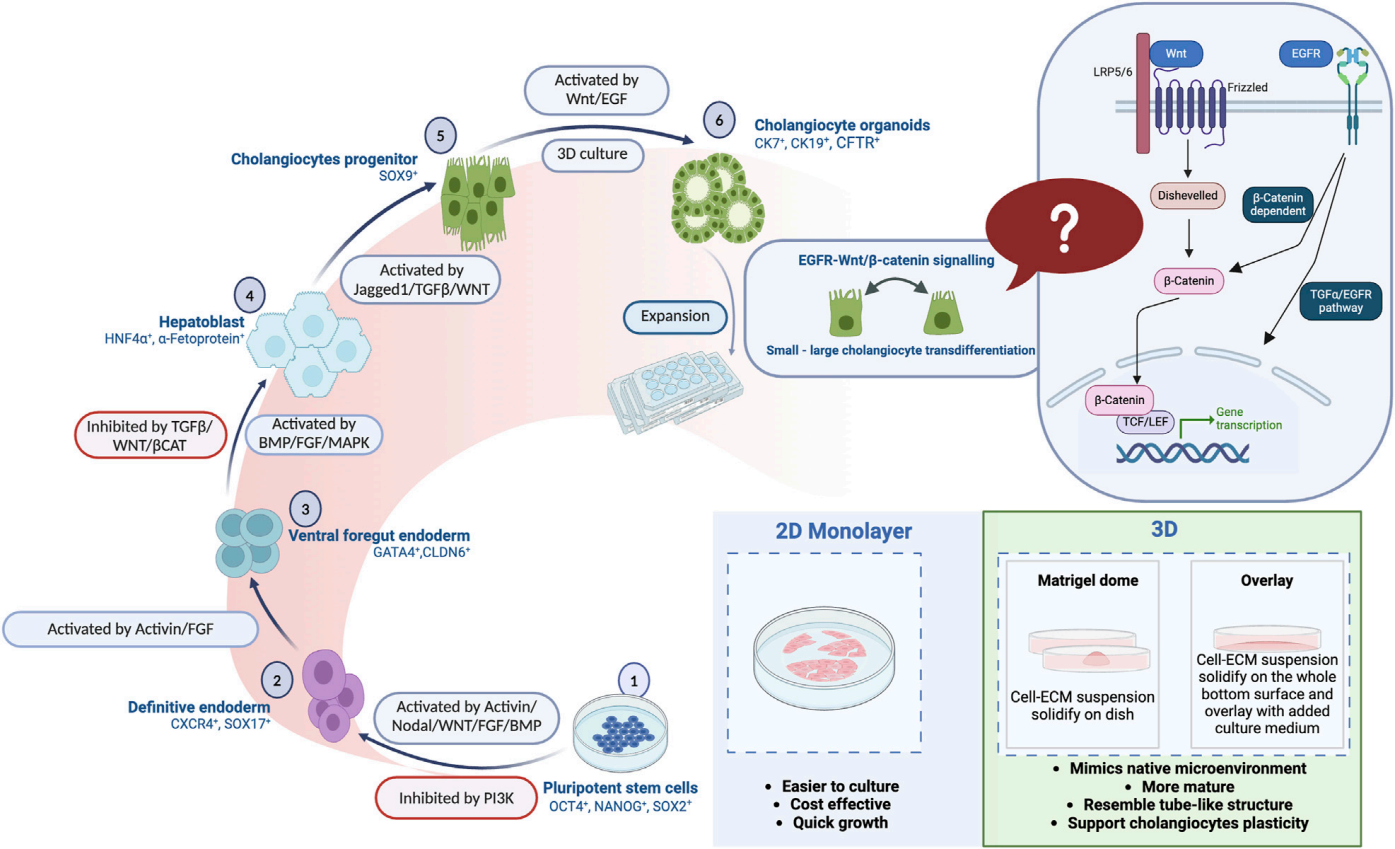
A comprehensive illustration is provided in the summary figure, depicting the differentiation stages of human pluripotent stem cells (PSCs) into mature cholangiocytes. Additionally, the figure highlights the advantages of both 2D and 3D organoid culture systems in facilitating the differentiation.

Several differentiation protocols have been reported for generating cholangiocytes from human ESCs or iPSCs using 2D or 3D organoid culture systems (Dianat et al., 2014; De Assuncao et al., 2015; Ogawa et al., 2015; Takayama et al., 2016; Sampaziotis et al., 2017a; Matsui et al., 2019; Luce and Dubart-Kupperschmitt, 2020; Ogawa et al., 2021; Jalan-Sakrikar et al., 2022). Consistent with our findings, some reports present that 3D culture systems provide a better culture niche for cholangiocyte maturation when compared to the 2D culture systems (Dianat et al., 2014; Sampaziotis et al., 2015a; Sampaziotis et al., 2017a; Ogawa et al., 2021). In advance of the previous works, our study provides a possible concept of small-large cholangiocyte transdifferentiation under 3D culture conditions with the illustration, highlighting the better potential in future clinical application (Figure 4B; Figure 7).

The generation of mature organoids is mediated by several factors, such as ECM, growth factors, cytokines, small molecules, signaling inhibitors, and coculture systems. Dianat et al. (2014) were the first to use OP9 stromal cells, in addition to growth factors such as HGF, EGF, and TGF-β1, for the differentiation of human PSCs and HepaRG into cholangiocyte-like cells (CLCs) with characteristics similar to the intrahepatic cholangiocytes that line the canals of Hering. OP9 stromal cells are line of murine stromal cells that are often used as feeder cells in cell culture systems for the differentiation of various cell types, including cholangiocyte differentiation by activating the NOTCH signaling (Ogawa et al., 2015; Ogawa et al., 2021). This approach aimed to mimic the *in vivo* developmental process of cholangiocytes (Dianat et al., 2014; Xu and Zhang, 2018). Although OP9 stromal cells are useful for studying various biological processes, their clinical utilization can present challenges related to species compatibility, safety, and regulatory compliance.

For research findings to be more feasibly translated into clinical applications, clinically relevant cell culture systems and models that are based on human-derived or human-compatible components should be developed and used. Sampaziotis et al. developed a robust protocol to generate cholangiocytes from human PSCs by using a chemically defined medium. They established an organoid model of several cholangiopathies, including cystic fibrosis (Sampaziotis et al., 2015a; Ogawa et al., 2015; Sampaziotis et al., 2017a). However, their protocol only generated CLCs that mimic intrahepatic cholangiocytes (Sampaziotis et al., 2017a; Ogawa et al., 2021).

Primary cholangiocyte organoids have been shown to have therapeutic effects aiding in the reconstruction of the bile duct in mice (Sampaziotis et al., 2017b) and in humans (Sampaziotis et al., 2021). The goals of regenerative medicine can be categorized in accordance with the 3R paradigm of replacement, regeneration, and rejuvenation (Nelson et al., 2008; Jalan-Sakrikar et al., 2023). Primary cholangiocytes derived from intrahepatic cholangiocytes transform into and rescue intrahepatic cholangiocytes after being transplanted into the intrahepatic bile duct and *vice versa*, supporting the concept of small-large cholangiocyte transdifferentiation according to the environment (Sampaziotis et al., 2021). Several studies have described that Wnt signaling may be responsible for intrahepatic and extrahepatic cholangiocyte transdifferentiation under certain conditions (Graffmann et al., 2018; Fried et al., 2020; Verstegen et al., 2020).

EGF has been reported to play a role in liver regeneration by replenishing damaged hepatocytes (Knight et al., 2019). The EGF/EGFR signaling pathway is known to regulate cell proliferation, differentiation, and survival. Additionally, EGF promotes the transdifferentiation of cholangiocytes into hepatocyte-like cells expressing hepatocyte-specific markers, such as albumin and cytokeratin 18 (Cerec et al., 2007; Verstegen et al., 2020). EGF also interacts with the Wnt/β-catenin signaling pathway, which is involved in liver development and regeneration (Nejak-Bowen and Monga, 2011). In pathological conditions like cholestasis and fibrosis, EGF may impact the balance between cholangiocytes and hepatocytes by modulating β-catenin activity (Okabe et al., 2016; Russell and Monga, 2018; Lee et al., 2010).

Wnt signaling is known to regulate hepatobiliary regeneration post-cholestatic injury through β-catenin-dependent and -independent mechanisms. The absence of β-catenin in liver cells (hepatocytes and cholangiocytes) is associated with abnormal bile duct structure, characterized by enlarged, twisted canaliculi and the absence of microvilli (Yeh et al., 2010). Furthermore, the inhibition of Wnt secretion from cholangiocytes leads to reduced cholangiocyte proliferation and transdifferentiation (Okabe et al., 2016; Knight et al., 2019).

In our study, the addition of EGF during the final stage of 3D cholangiocyte differentiation enhanced the maturation and transdifferentiation of cholangiocytes, as supported by RT-qPCR analysis, immunofluorescence staining to evaluate the core biliary markers and region-specific marker of intrahepatic and extrahepatic cholangiocyte (Figure 6). We observed a significant increase in the expression of genes associated with core biliary markers (*SOX9*, *CK19*, *CK7*), intrahepatic markers (*YAP1*, *JAG1*), and extrahepatic markers (*AQP1*, *MUC1*) upon EGF treatment (Figure 6A).

Additionally, we assessed the impact of EGF on 3D cholangiocyte morphology and CFTR protein expression (Figure 6B). Furthermore, compared to the control group, EGF treatment significantly upregulated genes associated with canonical Wnt signaling (*LGR5*) and non-canonical Wnt signaling (*NKCC1*), as well as the biliary progenitor gene, *NOTCH2* (Supplementary Figure S7). These findings are particularly intriguing as canonical and non-canonical Wnt signaling pathways have distinct impacts on the gene expression profiles and functional maturity of the cholangiocytes. Canonical Wnt signaling promotes stem cell-like characteristics, whereas non-canonical Wnt signaling fosters a more mature and functionally active cholangiocyte phenotype (Roos et al., 2020). Our findings provide initial evidence shedding light on the role of EGF in the maturation and proliferation of 3D cholangiocyte organoids in relation to Wnt signaling and *NOTCH2*. The cross-talk between these pathways may have an impact on the development and function of cholangiocytes, playing a significant role in liver regeneration and disease (Morell and Strazzabosco, 2014; Banales et al., 2019). Further comprehensive *in vitro* and *in vivo* studies are warranted to validate the concept of cholangiocyte transdifferentiation.

Other factors, such as the extracellular matrix (ECM) in 3D culture conditions, play a crucial role in supporting the concept of small-large cholangiocyte transdifferentiation and facilitating the mature and functional differentiation of cholangiocytes from human pluripotent stem cells *in vitro* (Sampaziotis et al., 2021). Matrigel, which consists of ECM components like laminin, collagen IV, entactin, and heparan sulfate proteoglycan, is commonly used, particularly for collagen I, XVIII, VI, and III (Hughes et al., 2010; Yee et al., 2022; Zhao et al., 2022). While Matrigel promotes organoid growth, its naturally heterogeneous and poorly defined composition limits control over the biochemical and biophysical cues crucial for enhancing organoid cultures (Spagnol et al., 2023). Our initial findings indicate that compared to laminin and Matrigel hESC-qualified matrix (Corning, 354277), the reduced growth factor Matrigel could enhance the differentiation of human pluripotent stem cells towards cholangiocytes (see Supplementary Figure S8), underscoring the significant role of specific ECM components in enhancing the differentiation of human pluripotent stem cells into cholangiocyte organoids.

## Conclusion

In this study, we established a robust, feeder-free, serum-free, cost-effective protocol for producing functional cholangiocytes from human iPSCs and ESCs that had particular success when used with a 3D culture system. This distinctive 3D culture platform generates mature cholangiocytes exhibiting high plasticity with tube-like structures, as well as facilitating small-large cholangiocyte transdifferentiation potentially regulated by the EGF-Wnt signaling pathway (Figure 7). The findings of this research could serve as a valuable reference for advancing cell-based therapies for cholangiopathies.

## Data Availability

The original contributions presented in the study are included in the article/Supplementary Material, further inquiries can be directed to the corresponding authors.

## Abbreviations

hPSC, human pluripotent stem cells; iPSC, induced pluripotent stem cells; ESC, embryonic stem cells; DE, definitive endoderm; VFE, ventral foregut endoderm; HB, hepatoblast; CP, cholangiocyte progenitor; CO, cholangiocyte organoid.
