# Supplementary material for: 3D organoid cultivation improves the maturation and functional differentiation of cholangiocytes from human pluripotent stem cells
Source: Front Cell Dev Biol. 2024 Jul 8;12:1361084. doi: 10.3389/fcell.2024.1361084 (PMC11260683; doi:10.3389/fcell.2024.1361084)
Supplement: Supplementary file 2 [file DataSheet1.pdf]

Supplementary Figures and Tables

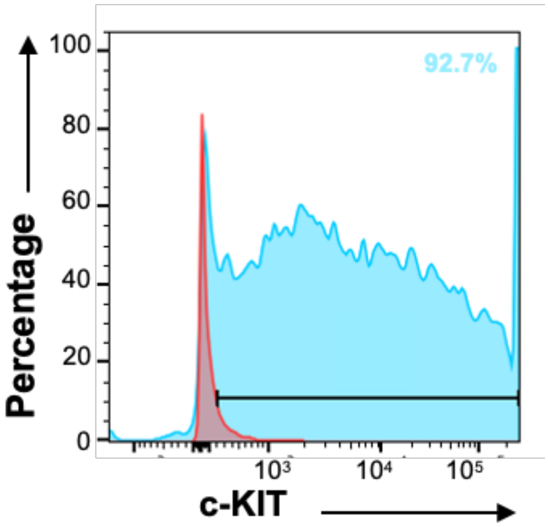

Supplementary Figure S1: Flow cytometry revealed that 92.7% of DEs were c-KIT^{+} at day 4 of differentiation.

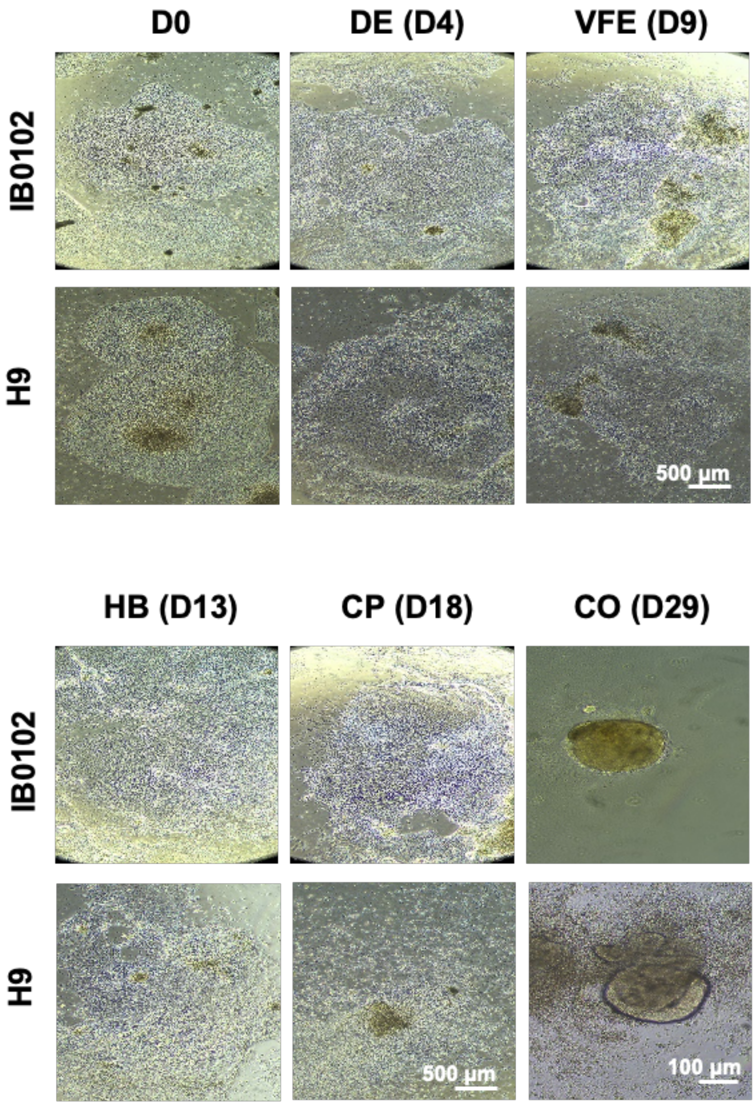

Supplementary Figure S2: Representative images of each stage of differentiation from another clone of human iPSC (IB0102) and ESC (H9).

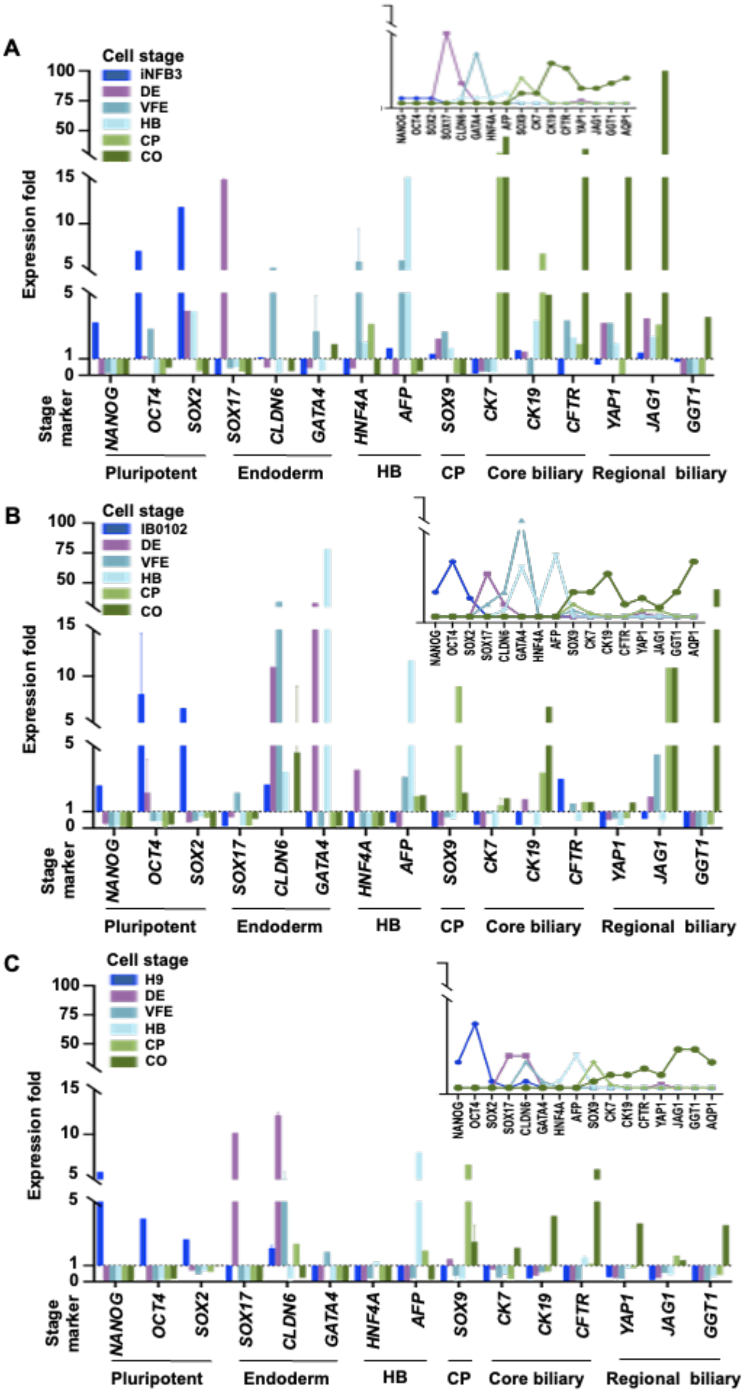

Supplementary Figure S3: RT-qPCR panel throughout the cholangiocyte organoid differentiation from 2 hiPSC clones and 1 hESC cell line. **(A)** RT-qPCR shows a significant increase in markers on each differentiation stage from iNFB3. **(B)** RT-qPCR shows a significant increase in markers on each differentiation stage from IB0102. **(C)** RT-qPCR shows a significant increase in markers on each differentiation stage from H9.

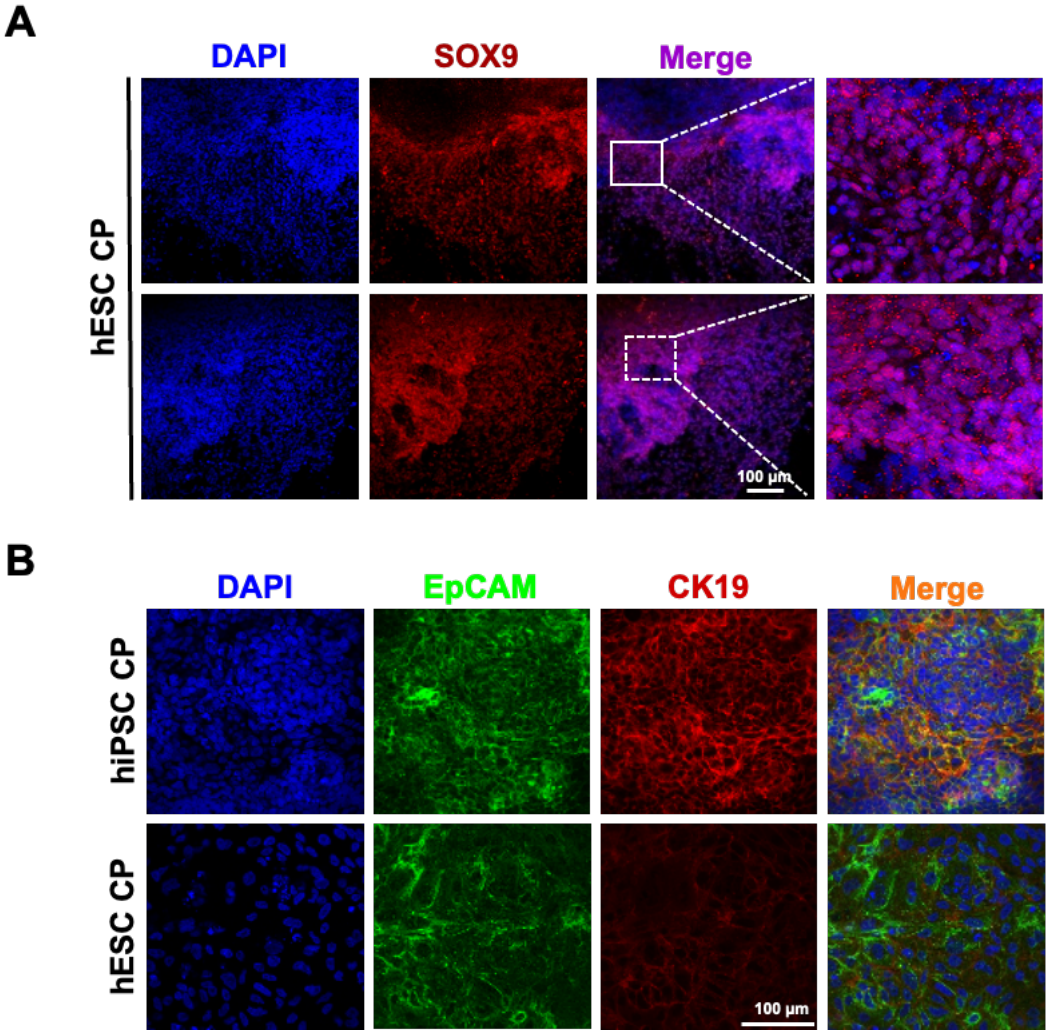

Supplementary Figure S4: Characterization of cholangiocyte progenitor (CP) stage. **(A)** hPSC-derived CP at day 18 of differentiation shows positive for early cholangiocyte marker SOX9. **(B)** CPs under prolonged 2D culture showed positive for EpCAM and CK19 at day 28 of differentiation.

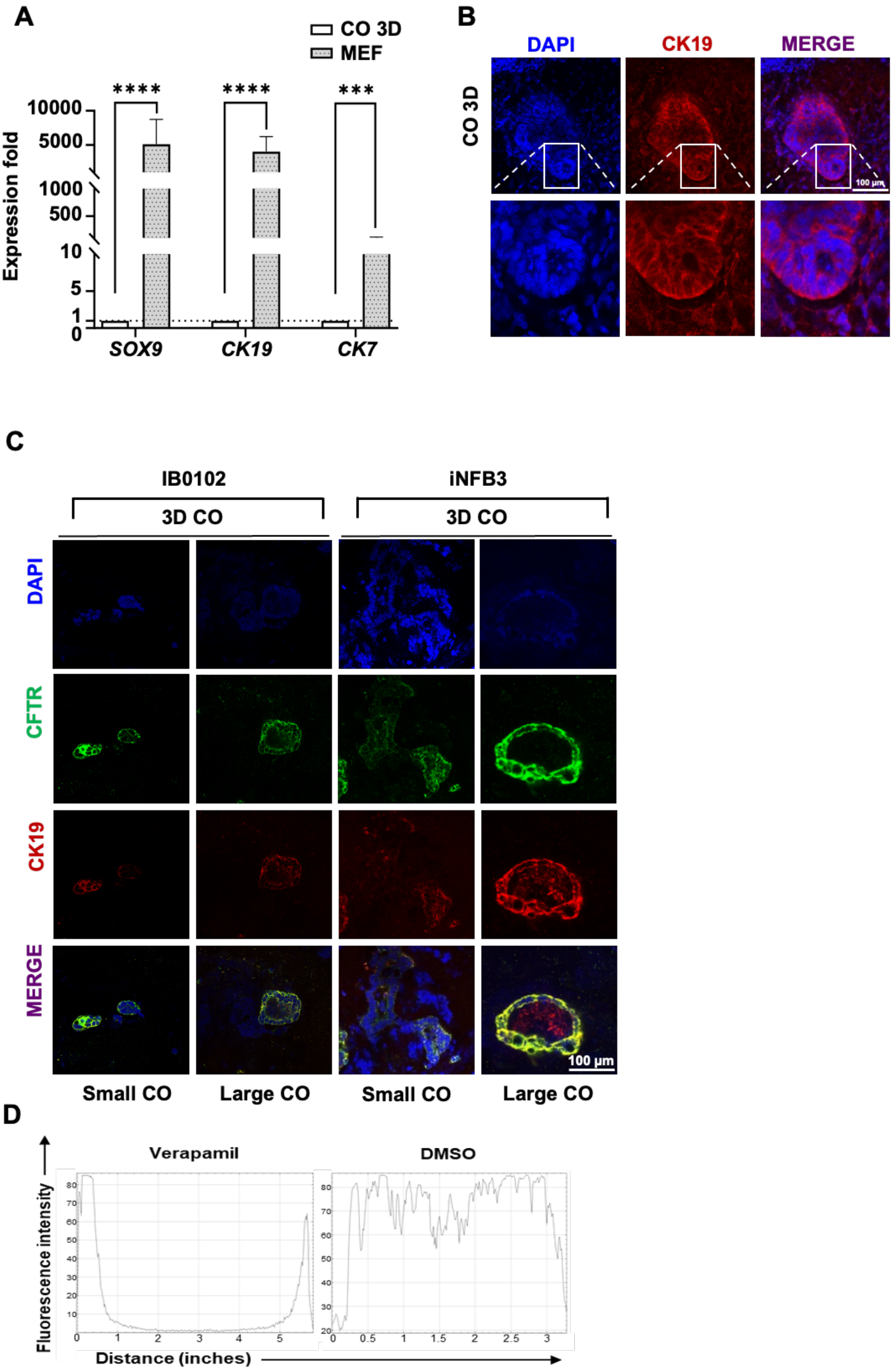
A
CO 3D
MEF
Expression fold
10000
5000
1000
500
10
5
1
0
****
****
***
SOX9
CK19
CK7
B
DAPI
CK19
MERGE
CO 3D
100 μm
C
IB0102
iNFB3
3D CO
3D CO
DAPI
CFTR
CK19
MERGE
100 μm
Small CO
Large CO
Small CO
Large CO
D
Verapamil
DMSO
Fluorescence intensity
Distance (inches)

Supplementary Figure S5: Protocol validation using a human ESC line, H9, and another human iPSC line, IBMS-iPSC-01-02. (A) RT-qPCR of hESC-derived CO under 3D culture shows that the mature cholangiocyte markers, SCTR, CFTR, and CK19, were significantly increased compared to MEF. (B) Immunofluorescence staining of human ESC-derived COs shows a positive for CK19. (C) IF staining shows the morphology of COs derived from IBMS-iPSC-01-02 and iNFB3, which also indicates positive for CFTR and CK19 and has the lumen inside the organoid. (D) Rhodamine-123 fluorescence intensity after Verapamil and DMSO treatment on human PSC-derived COs. Rhodamine-123 fluorescence quantification was performed by using ImageJ (Schneider et al., 2012). MEF, mouse embryonic fibroblast; DMSO, dimethyl sulfoxide.

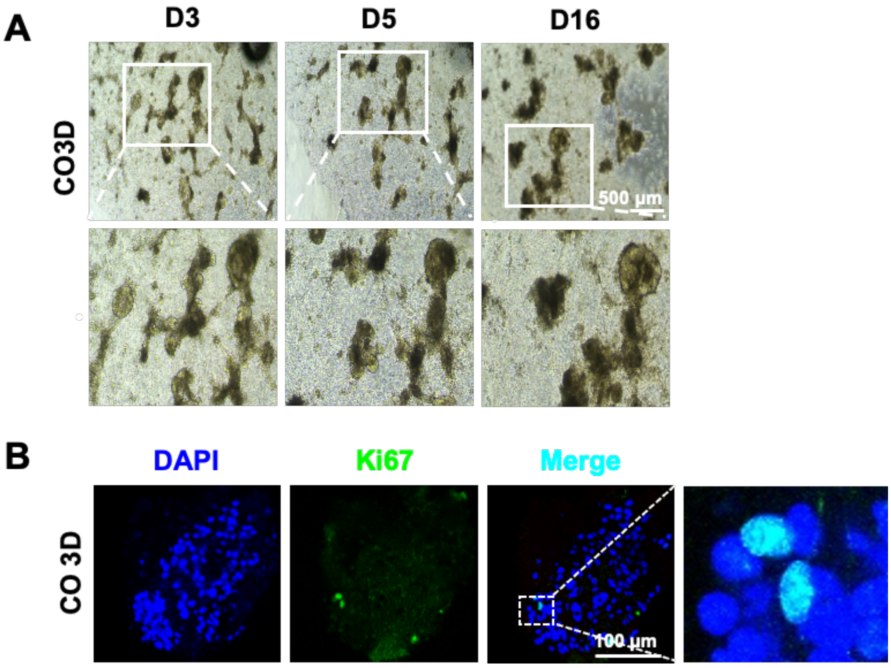

Supplementary Figure S6: Expansion of human iPSC-derived CO under 3D culture. **(A)** human iPSC-derived COs forming tubular-like structures under 3D culture expansion (P2-3). **(B)** IF staining for Ki67 shows that in long-term culture after passaging, human iPSC-derived COs decrease their proliferation capacity, which is evaluated by the decrease of Ki67 positive cells.

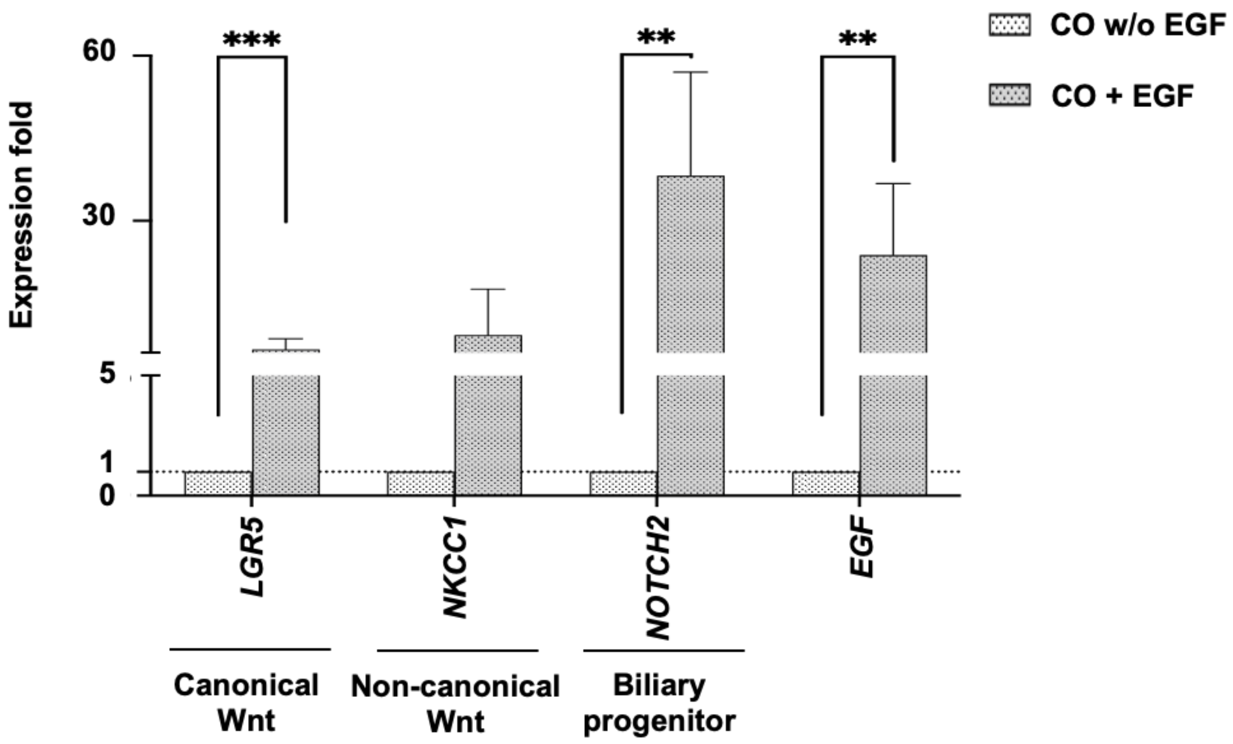

Supplementary Figure S7: Canonical and non-canonical Wnt siganling target genes, along with *NOTCH2*, were upregulated in CO in the presence of EGF.

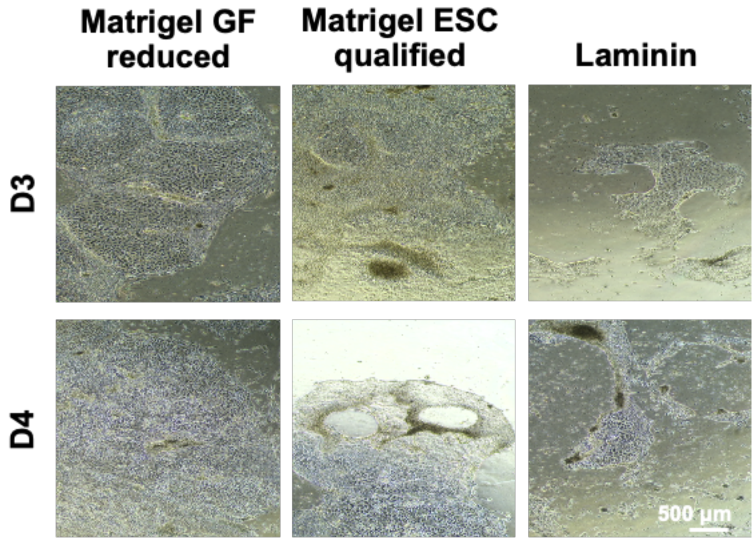

Supplementary Figure S8: Preliminary results show the cell morphology on Day 3 (D3) and Day 4 (D4) of cholangiocyte differentiation from hPSCs. The use of Matrigel with reduced growth factors (GF) resulted in noticeable changes in cell morphology compared to Matrigel specifically qualified for embryonic stem cells (ESC) or laminin. Additionally, the number of cells in the laminin group was significantly reduced.

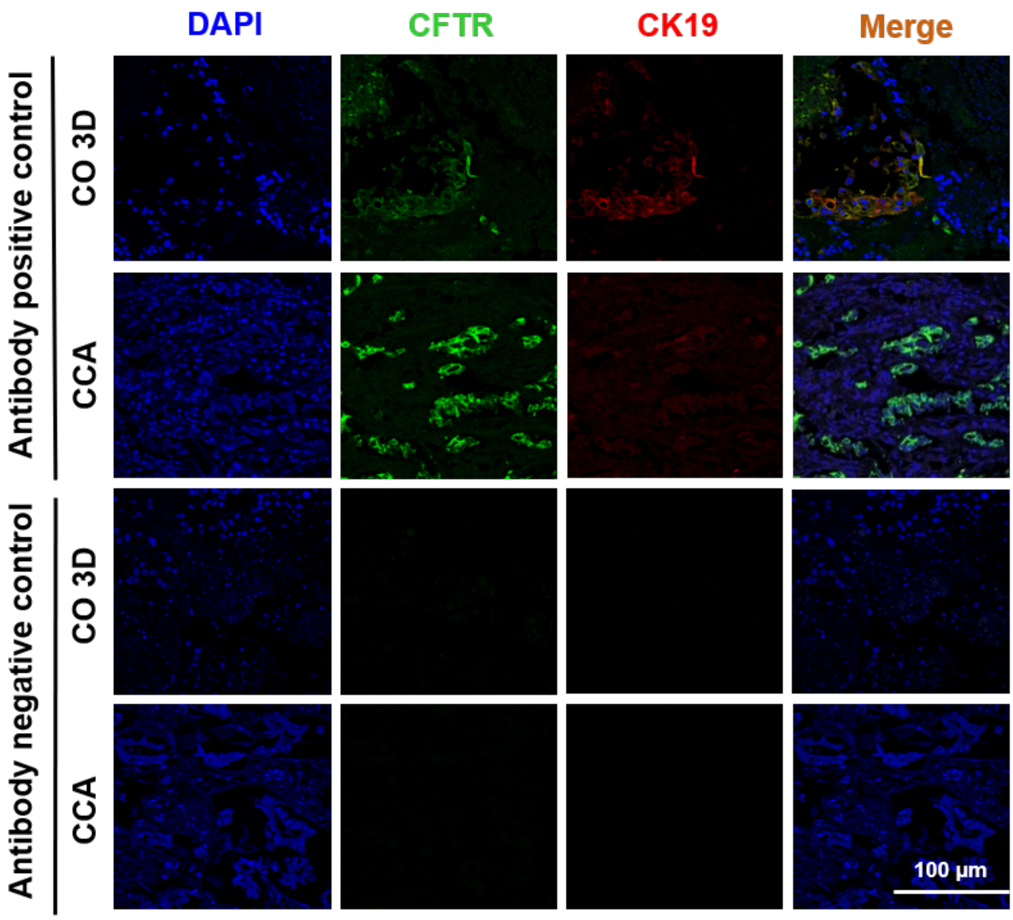

Supplementary Figure S9: Representative images of antibody control for immunofluorescence staining. CCA, cholangiocarcinoma tissue section.

Supplementary Table S1: List of reagents used in cholangiocyte differentiation from hPSCs in 2D and 3D culture system.

Stage	Day of differentiation	Supplement	Cat. Number	Brand	Concentration
hPSCs ➔DE	D1	Activin A	338-AC	R&D Systems	100 ng/mL
		bFGF	F0291	Sigma-Aldrich	80 ng/mL
		BMP-4	CYT-361	Prospec	10 ng/mL
		LY29002	HY-10108	MedChemExpress	10 µM
		CHIR99021	HY-10182	MedChemExpress	3 µM
	2	Activin A	R2625	Sigma-Aldrich	100 ng/mL
		bFGF	F0291	Sigma-Aldrich	80 ng/mL
		BMP-4	CYT-361	Prospec	10 ng/mL
		LY29002	HY-10108	MedChemExpress	10 µM
	D3-D4	Activin A	338-AC	R&D systems	100 ng/mL
		bFGF	F0291	Sigma-Aldrich	80 ng/mL
DE ➔ VFE	D5-D8	Activin A	R2625	Sigma-Aldrich	50 ng/mL
VFE ➔HB	D9-D13	SB-431542	HY-10431	MedChemExpress	10 µM
		BMP-4	CYT-361	Prospec	50 ng/mL
HB ➔ CP	D14-D18	FGF10	PHG0204	Invitrogen	50 ng/mL
		Activin A	338-AC	R&D systems	50 ng/mL
		Retinoic acid	R2625	Sigma-Aldrich	3 µM
CP ➔ CO	D19	EGF	PHG0311L	Gibco	10 ng/mL
		Y-27632	HY-10583	MedChemExpress	10 µM
	D20-D28	EGF	PHG0311L	Gibco	10 ng/mL

Supplementary Table S2: Protocol of cholangiocyte differentiation from hPSCs in 2D and 3D culture systems (Adopted and modified from prior studies).

<table>
<tr><th colspan="2">Differentiation Step</th><th colspan="2">Protocol Details</th></tr>
<tr><td colspan="2">hPSC Seeding, D0</td><td>2D</td><td>3D</td></tr>
<tr><td rowspan="2">1</td><td rowspan="2">Maintenance of hPSCs Colonies</td><td colspan="2">Cultivate the human embryonic stem cells (hESCs) and induced pluripotent stem cells (hiPSCs) using proprietary “Primary ES Medium” and supplemented with 12 ng/ml bFGF and 0.5X penicillin-streptomycin.</td></tr>
<tr><td colspan="2">Renew medium daily to sustain optimal growth conditions.</td></tr>
<tr><td>2</td><td>Preparation of culture Plates</td><td colspan="2">Pre-coat 12-well culture plates with 3% Matrigel for 30-60 minutes before cell seeding to promote attachment and support stem cell maintenance.</td></tr>
<tr><td>3</td><td>Cell seeding</td><td colspan="2">Seed 1-1.5 ml of hPSCs medium onto each well of the pre-coated culture plates.</td></tr>
<tr><td></td><td></td><td colspan="2">Ensure proper clump size during mechanical dissociation to facilitate subsequent differentiation.</td></tr>
<tr><td>4</td><td>Pre-differentiation Cultivation</td><td colspan="2">Allow cells to acclimate and proliferate for two days prior to commencing the differentiation process.</td></tr>
<tr><td colspan="4">hPSCs to Definitive Endoderm (DE), D1-D4</td></tr>
<tr><td>5</td><td>Day 1</td><td colspan="2">Replace hPSCs cultivation medium with cholangiocyte differentiation medium consisting of RPMI 1640 supplemented with 1X B27 supplement, and:
• 100 ng/mL activin A
• 80 ng/mL bFGF
• 10 ng/mL BMP-4
• 10 µM LY29002
• 3 µM CHIR99021
to induce the DE specification.</td></tr>
<tr><td>6</td><td>Day 2</td><td colspan="2">Continue the differentiation with differentiation medium as the same as day 1 without the CHIRR99021:
• 100 ng/mL activin A
• 80 ng/mL bFGF</td></tr>
</table>

		• 10 ng/mL BMP-4<br>• 10 µM LY29002<br>to sustain DE commitment and suppress alternative lineage differentiation pathways.	
**7**	Day 3-4	Replace the differentiation medium with the RPMI/B27 medium supplemented with only high dose of:<br>• 100 ng/mL activin A<br>• 80 ng/mL bFGF<br>to support endodermal lineage maturation and monitor expression of endodermal markers.	
**DE to Ventral Foregut Endoderm (VFE), D5-D8**			
**8**	Day 5-8	Cultivate cells in RPMI/B27 medium supplemented with lower 50 ng/mL activin A to steer DE toward a VFE fate, fostering further differentiation and lineage specification.	
**VFE to Hepatoblast (HB) or Hepatic Bud Progenitor, D9-D13**			
**9**	Day 9-13	Replace the differentiation medium with RPMI/B27 medium supplemented with:<br>• 10 µM SB-431542<br>• 50 ng/mL BMP-4<br>to facilitate HB progenitor specification and evaluate expression of key hepatic lineage markers.	
**HB to Cholangiocyte Progenitors (CP), D14-D18**			
**10**	Day 14-18	Replace the differentiation medium RPMI/B27 medium supplemented with:<br>• 50 ng/mL FGF10<br>• 50 ng/mL activin A<br>• 3 µM retinoic acid<br>to drive differentiation toward cholangiocyte progenitors while monitoring the expression of cholangiocyte lineage markers.	
**CPs and Generation of Cholangiocyte Organoids (COs), D19 -D20**			
**11**	Day 19	Remove the medium and wash the cells with PBS. Detach the cells by using TrypLE Select CTS and	Detach cells using PBS and cell dissociation buffer, followed by mechanical dissociation as necessary.

		centrifuge by using 400G to retain the cells. Culture cells on the T75 flask for one day.	Collect dissociated cells, and resuspend in Matrigel/WE medium supplemented with:<br>• 10 ng/mL EGF<br>• 10 µM Y-27632
		On the next day, take the cells cultured on the T75 dish and centrifuge for 400g in 5 minutes. Cultured the cells in WE medium supplemented with:<br>• 10 ng/mL EGF<br>• 10 µM Y-27632	Seed the cells/Matrigel suspension in the in pre-prepared 12-well plates. Allow the Matrigel dome to solidify for approximately 10 minutes. Next, place the Matrigel dome in an inverted position wihin a 37°C incubator and incubate for around 20 minutes to facilitate cell floating inside the dome.<br>Put the Matrigel dome to the 37oC incubator on inverted position and wait for 15-20 minutes to let the cells floating on the Matrigel dome.<br>Once the Matrigel dome has solidified, add the WE medium supplemented with:<br>• 10 ng/mL EGF<br>• 10 µM Y-27632
**12**		Seed the cells on another new 3% Matrigel coated dish with WE medium supplemented with 10 ng/mL EGF.	Culture organoids in supplemented WE medium with:<br>• 10 ng/mL EGF<br>• 10 µM Y-27632
**Maturation of CO Organoids, D21-D28**			
**13**	Day 21-28	Renew the medium with WE medium supplemented with EGF (10 ng/mL) every two days until day 28	Perform bi-daily medium changes using WE medium supplemented with EGF (10 ng/mL) to support organoid maturation until day-28.

Supplementary Table S3: List of RT-qPCR Primer Sequence

Real-time quantitative PCR primers			
**Gene**	**Accession**	**Forward Primer**	**Reverse Primer**
OCT4	NM_002701	5'-CAACTCCGATGGGGCCT-3'	5'-CTTCAGGAGCTTGGCAAATTG-3'
NANOG	NM_024865	5'-CCTGTGATTTGTGGGCCTG-3'	5'-GACAGTCTCCGTGTGAGGCAT-3'
SOX2	NM_003106	5'-GTATCAGGAGTTGTCAAGGCAGAG-3'	5'-TCCTAGTCTTAAAGAGGCAGCAAA-3'
SOX17	NM_022454.4	5'-ACGCTTTCATGGTGTGGGCTAAG-3'	5'-CCAAGGTGATCCTCTTCTGCTTCAG-3'
GATA4	NM_001308093.3	5'-GAAAACGGAAGCCCAAGAACC-3'	5'-AGACATCGCACTGACTGAGAACG-3'
HNF4α	NM_010137	5'-GGTGTCCATACGCATCCTTGAC-3'	5'-GCCGCTTGATCTTCCCTGGAT-3'
SOX9	NM_000346.4	5'-AGGAAGCTCGCGGACCAGTAC-3'	5'-GGTGGTCCTTCTTGTGCTGCAC-3'
CK19	NM_002276.5	5'-AGCTAGAGGTGAAGATCCGCGA-3'	5'-GCAGGACAATCCTGGAGTTCTC-3'
CK7	NM_005556.4	5'-TGTGGATGCTGCCTACATGAGC-3'	5'-AGCACCACAGATGTGTCGGAGA-3'
AFP	NM_001134	5'-ACAGAGGAACAACTTGAGGCTGTC-3'	5'-AGCAAAGCAGACTTCCTGTTCCTG-3'
ALB	NM_000477	5'-GTGAAACACAAGCCCAAGGCAACA-3'	5'-TCAGCCTTGCAGCACTTCTCTACA-3'
GGT1	NM_053840.2	5'-TGACGTACCACCGCATCGTAGA-3'	5'-CAGCGAAGAACTCGGAGGTCAT-3'
SCTR	NM_002980	5'-AGAGGACGCTATGGAGATCCAG-3'	5'-AACTCACGGAGGTGCCATTGCT-3'
CFTR	NM_000492	5'-AGGACTATGGACACTTCGTGCCTT-3'	5'-ATTTGGAACCAGCGCAGTGTTGAC-3'
AQP1	NM_007472	5'-TCTTCCGTGCCCTCATGTA-3'	5'-CAAGCGAGTTCCCAGTCAG-3'
CLDN6	NM_021195	5'-GTGGAAGGTGACCGCTTTCATC-3'	5'-CAGCAGTGAGTCGTACACCTTG-3'
MUC1	NM_002456	5'-GTGCCCCCTAGCAGTACCG-3'	5'-GACGTGCCCCTACAAGTTGG-3'
JAG1	NM_000214	5'-TGCTACAACCGTGCCAGTGACT-3'	5'-TCAGGTGTGTCGTTGGAAGCCA-3'
YAP1	NM_006106	5'-TGTCCCAGATGAACGTCACAGC-3'	5'-TGGTGGCTGTTTCACTGGAGCA-3'
GGT1	NM_005265	5'-TGACGTACCACCGCATCGTAGA-3'	5'- CAGCGAAGAACTCGGAGGTCAT-3'
EGF	NM_001963	5'-TGCGATGCCAAGCAGTCTGTGA-3'	5'-GCATAGCCCAATCTGAGAACCAC-3'
NOTCH2	NM_024408	5'-GTGCCTATGTCCATCTGGATGG-3'	5'-AGACACCTGAGTGCTGGCACAA-3'
LGR5	NM_003667	5'-CCTGCTTGACTTTGAGGAAGACC-3'	5'-CCAGCCATCAAGCAGGTGTTCA-3'
NKCC1	NM_001046	5'-CCTCTACACAAGCCCTGACTTAC-3'	5'-CGTGAGTTTGGAGCACCTGTCA-3'

Supplementary Table S4: List of antibodies used in this study

Protein	Assay	Ab Cat. No.	Company	Origin	Dilution	Incubation Period	Protein Size (kDa)
Oct-4	IF<br>WB	sc-8628	Santa Cruz Biotech	goat	1:200<br>1:500	overnight, 4°C	43
SOX2	IF<br>WB	ab97959	abcam	rabbit	1:300<br>1:500	overnight, 4°C	34
NANOG	WB	ab21624	abcam	rabbit	1:500	overnight, 4°C	34
CXCR4	FC<br>IF	ab124824	abcam	rabbit	1:100<br>1:500	1 hr, RT<br>overnight, 4°C	39
β-ACTIN	WB	sc-47778	Santa Cruz Biotech	mouse	1:500	overnight, 4°C	43
c-KIT	FC	sc-365504	Santa Cruz Biotech	mouse	1:100	1 hr, RT	120
SOX-9	IF	sc-20095	Santa Cruz Biotech	rabbit	1:200	overnight, 4°C	65
CFTR	IF	sc-376683	Santa Cruz Biotech	mouse	1:200	overnight, 4°C	165
AFP	FC<br>IF	E-AB-22033	Elabscience	mouse	1:300	1 hr, RT<br>overnight, 4°C	70
CK19	IF	E-AB-40479	Elabscience	rabbit	1:300	overnight, 4°C	44
SOX17	FC<br>IF	AF1924	R&D systems	goat	1:200<br>1:500	1 hr, RT<br>overnight, 4°C	55
EpCAM	FC<br>IF	#2929	Cell Signaling	mouse	1:300<br>1:500	1 hr, RT<br>overnight, 4°C	40
Ki-67	IF	ab15580	abcam	rabbit	1:200	overnight, 4°C	320

IF: immunofluorescence staining; WB: western blotting; FC: flow cytometry
